# The impact of medical service on the return behavior: A city-level study in China

**DOI:** 10.3389/fpubh.2022.1009454

**Published:** 2022-10-24

**Authors:** Mingming Meng, Zheng Wang, Ji'an Yu

**Affiliations:** ^1^School of Economics and Management, Beijing Forestry University, Beijing, China; ^2^Center of Healthcare Management, School of Economics and Management, Tsinghua University, Beijing, China

**Keywords:** medical service, return behavior, health status, health education, rural household registration, new rural cooperative medical system

## Abstract

Due to the constraints of the rural-urban household registration systems, the migrants of China currently receive varying degrees of medical services. The fact that many migrants choose to return to their hometowns due to the inequality in medical care has been a social phenomenon. Using data from the 2017 China Migrant Dynamic Survey (CMDS), this paper explores the effect of medical services on population migration. Probit regression analysis method was utilized to examine the relationship between medical service level (MSL) and medical service improvement (MSI) and return behavior (RB), as well as the interaction effect between MSL and MSI, and the moderating effect of health status (HS) and health education (HE). Multiple heterogeneity tests were performed. Grouping regressions were conducted using rural household registration (RHR), grouping regressions were conducted using new rural cooperative medical system (NRCMS), and multinomial Probit regressions were conducted using migration distance and age factors. The following findings were obtained. First, when MSL is low but MSI is high in the locality of household registration, the return probability of migrants will increase. MSL also has a positive interaction effect with MSI, and they jointly increase the return probability of migrants; Second, HS and HE have a positive moderating effect on the relationships between MSL and RB and between MSI and RB; Third, heterogeneity analysis indicates that the migrants with RHR or the migrants not covered by the NRCMS are more prone to return due to the reason of medical service. In addition, the analysis also shows that middle-aged and older people who return across provinces have the highest tendency to return due to medical services and young people have the lowest propensity to return across and within provinces. The study could help local governments change their public medical care policies and close the gap between medical services in different areas. As a result, it is necessary to understand population migration trends and promote New Urbanization Strategies.

## Introduction

Since the reform and opening-up in China, large-scale cross-regional population migration has constantly been reshaping the spatial pattern distribution of population. Due to the uneven regional economic development and loosened household registration policy, nationwide waves of population migration have emerged. According to the data of the 7th National Population Census released by the National Bureau of Statistics in 2021, the total migrants in China have grown significantly to 375.82 million in 2020 from 221.43 million in 2010, representing an annual growth rate of 6.97%. With industrial restructuring and upgrading in the coastal regions and the shift of labor-intensive industries toward central and western regions where considerable economic development has been gained, the rural and urban employment landscape has drastically changed, and the return of the migrants to their hometowns have become an increasingly widespread phenomenon. In 2014, the Chinese government formulated the New Urbanization Development Plan to encourage the migrants to transfer their rural household registration to cities and towns, especially small and medium-sized cities closer to their homes. This initiative has successfully promoted the large-scale return of the migrants. According to the China Migrants Development Report, about 22.8% of the migrants chose to return in 2017, and more than 70% of the returning population said they did not want to go back out (1). Especially in recent years, many rural laborers from central and western regions have chosen to return to developed coastal areas due to the impact of the COVID-19 epidemic, and the population migration shows the co-existence trend of outflow and inflow.

Population migration has drawn increasing scholarly interest and relevant studies mainly focus on settlement intention (2, 3), income level (4), health (5), social integration (6), and other aspects of the migrants. The population migration often exhibits three patterns: settling in inflow region, continuous migration and return migration. With improvements in welfare associated with China's RHR, advancements in rural area construction and the increasing effect of the market on resource allocation, return migration has become an important option for population migration (7). Earlier theories on return migration included neoclassical microeconomics, the new economics of migration and social network theory. Neoclassical micro economists, represented by Todaro (8), maintain that return migration is a decision made by migrants after weighing the difference between the maximum expected return and migration cost; supporters of the new economics of migration assert that return migration is the behavior of migrants aiming at maximizing their household welfare (9). And the social network theory emphasizes that the social connections and social network in the outflow region constitute the main factor driving migrant return (10). Research on return migration in China began in the 1990s, and the unique rural land system in China has created conditions for return migration. Differing from the universal term of migrants in western theories, the return migration in China's context involves more finely divided individuals like the new generation of migrant workers, college students from the second generation of peasant families and returning entrepreneurs. Chinese scholars have mainly focused on the differentiated return intentions of the migrants caused by policy, social, environmental and economic factors (7).

Currently, scholars have studied on the topic of medical services for migrants. For example, Ismayilova et al. (11) mainly studied the relationship between migration status, migration patterns and access to health care among Kazakhstan's labor migrants. Kim et al. (12) focused on the health utilization patterns and healthcare needs among Korean expatriates in Vietnam, Cambodia, and Uzbekistan. Studies by Vaalavuo and Sihvola (13) found that the impact of specialized health care services on population migration in Finland. Snyder and Wilson (14) conducted a study on the association between urban Aboriginal peoples' mobility and health service use in two distinct Canadian cities. Gu et al. (15) investigated the effect of the quantity and quality of health services on emergent migration in China. However, current studies on return migration have barely addressed medical services, and most studies have concentrated on return intentions. For example, based on a survey of African immigrant groups in Spain and Italy, De Haas and Fokkema (16) examined how social culture, economy, international connection and other factors affect return intentions of the migrants. Haug (17) examined the effect of social capital in the inflow region on international immigrants' return intentions. Li et al. (18) explored the relationship between familial factors and the return intentions of migrant workers. Studies by Gu et al. (19) found that factors influencing the return intentions of migrant workers in China to return home, including familial relationships, housing, society and space. Overall speaking, although the return intentions reflect and predicts, to some extent, the future return trend of the migrants, it does not represent the final return result (20). In fact, the emergence of return intentions is only the starting point, and RB is the final result of this process (21). Nonetheless, studies of RB are few except those by a few scholars like Leibbrand and Zhang (22, 23). Therefore, not only the topic of medical services has not been involved in the RB of migrants in previous studies, but also the returning problem has been mainly focused on return intentions and has not been involved in RB. In view of the deficiencies in the above studies, it is necessary to conduct an in-depth study on medical services and RB of migrants.

Considering that in China, migrants are characterized by low socioeconomic status, low socioeconomic status, labor-intensive jobs and a lack of convenient access to health insurance. In the meantime, medical services for the migrants have not been given enough attention. All of these have put medical services for the migrants in a disadvantageous position. Against the background of an increasing need for medical services, medical services in the original inflow regions are still highly exclusive to the migrants, where local residents are still prioritized in the distribution of rivalrous public goods (24). Under such conditions, constantly improving MSL in the city of their registered residence becomes more attractive to the migrants. Therefore, the following questions are raised: Does medical service cause the migrants to make return decisions and behavior? To what extent does it affect such decisions and behavior? How does this effect differ across individuals with different characteristics? It is difficult to answer these questions through traditional microscopic data research because return as a retrospective migration activity requires the acquisition of information about individuals' historical migration behaviors. Limited by data availability, the existing literature is mostly concentrated on theoretical analysis or exploration of the return intentions based on questionnaire-based surveys, without examining the reality of RB. This paper tries examine the effect of medical services on RB of the migrants and the moderating effect of HS and HE of the migrants, which could not only help gain an in-depth understanding of the patterns of population migration and promoting coordinated development, but help local governments timely adjust their public medical care policies and smoothen population return channels.

## Research hypothesis

The Law of the People's Republic of China on the Promotion of Basic Medical and Health Care, promulgated in 2019, explicitly points out that medical services fall in the category of medical and health services, which are disease prevention, diagnosis, treatment, nursing, rehabilitation and other services provided by adopting suitable drugs, appropriate technologies and suitable equipment. Based on the concept stated above, this paper decomposes medical services into MSL and MSI, which, respectively, mean the absolute value of medical service resources in one city area in a given year and the level of improvement of such medical service resources within a certain period of time. Moreover, defined from a spatial perspective, RB refers to a phenomenon that the migrants leave the city where the household registration is located during the process of migration, and then returns to the city where the household registration is located after a period of time (7). For the migrants in China, the restrictions of the household registration system make it impossible for them to truly enjoy the supporting medical services in the inflow region (25). An inability to obtain local household registration poses a huge barrier to accessing health care and insurance (26). The results of the Seventh National Census show that the inflow of migrants still mainly occurs in the more economically developed eastern region. Despite a high MSL, the large population base of the region has caused a limited MSL available per capita and indirectly resulted in relatively high medical service expenses. Although China has proposed to establish an instant settlement system for medical treatment in different places since 2010, under the localized management of medical insurance, there are often problems such as low reimbursement ratio, cumbersome procedures related to settlement, difficulty in information sharing, and pressure on fund advance, etc. Participating in medical insurance locally can save many reimbursement troubles for migrant people^1^. At the same time, in the Chinese context, even if a migrant is ill in a non-registered cities, he or she expects to be accompanied by a relative from afar, as opposed to the care of an hired caregiver. Kinship care is not just companionship when one is sick but also spiritual care. Because distance is a central factor in the spatial choices of migrants from a geographic perspective (27), the cost of companionship incurred away from home, family, and friends tend to increase with distance. Considering that migrants generally have low-income levels and higher financial insecurity (26), the scarcity of health insurance in inflow urban cities and the additional costs arising from off-site hospitalization and care, all of these reasons lead to migrants paying higher costs for health care services than local residents (28), and further enhance their desire for more affordable medical services. Hence, with barriers to accessing medical services in the target areas of migration, some of the migrants have to return home to seek more cost-effective medical services (29). Although a relatively lower MSL in central and western China, the higher convenience and affordable prices of medical services in their home regions still account for their choice to seek medical services in the city of registered residence. Additionally, with progress in implementing “The Medical Insurance System Covering the Whole Population,” the levels of basic medical insurance and public medical services targeting populations in less developed regions and rural areas have also been steadily improving. This has stimulated their willingness to return, causing increasing MSL to generate huge attractiveness to the migrants. To sum up, when MSL is relatively low but MSI is considerably large in cities of household registration, the migrants still tend to make the return decision and thus increase the return probability given their considerations of restrictions of the household registration system and financial factors. Therefore, the following hypotheses are proposed:

***H1: When MSL in the household city is low, but MSI is high, the return*** ***probability of migrants will increase*.**

After understanding the effects of MSL and MSI in cities of household registration, MSL and MSI also promote and influence each other and jointly promote the occurrence of RB. The low level of medical services in the region will encourage the local government to increase medical investment, which in turn will improve the medical service situation and enhance the quality of medical services, thereby increasing the probability of the migrants returning. Existing studies also confirm that province-level units with less developed public medical services should enhance their capacity in the area (30). With the promulgation of the Plan for Universal Medical Care during the “14^th^ 5-year Plan,” advancing the construction of urban-rural medical coordination system and enhancing the support for medical care in less developed regions have been recognized as priorities for governmental work. Continuous improvement in medical services also helps reverse the backwardness of the level of medical services in these regions. Presently, the need for medical services has been a fast ascent (31), forcing some migrants who sacrifice part of their medical service needs in the face of dual pressures from the household registration system and financial conditions to return home (25). If medical services in the city of registered residence continue to improve to a considerable extent, the city of registered residence will have a significant appeal to the migrants even if the absolute level of medical services still lags behind that of the more developed regions in the east. The above analysis indicates that the interactions between MSL and MSI increase the return probability of migrants, so the following hypotheses are proposed:

***H2: There is a positive interaction effect between MSL and MSI. The larger*** ***the interaction effect, the higher the return probability of migrants*.**

The relationship between the migrants and the state of their physical health is referred to by scholars as the “healthy migrant effect” (32). Studies show that the migrants enjoy an advantage of good health during their early stage of migration, but with progression in time, their health advantage gradually diminishes and factors like long working time, poor working conditions and barriers against accessing health care services in the inflow regions all contribute to the decline of the health of the migrants (33). Eventually, those with poor health are more likely to return home (34, 35). Long further points out that the returning behavior of the migrants as a result of health issues actually represents a contradiction between urban-rural medical resource distribution and the need for these resources, reflecting the role of the medical service situation in driving the return behavior of the migrants (36). In the meantime, migrants in poor health are more likely to experience worsening economic conditions due to the pressure of health care costs, making them more likely to be attracted to return by the relatively affordable health care services available in the city of registered residence. Therefore, migrants with poor health conditions are more likely to exhibit RB due to their inability to pay for and enjoy the medical services provided by their original inflow cities. Meanwhile, they are more included to choose the return option when MSL improves in the city of registered residence. The above analysis shows poor health conditions have a moderating effect on the relationships between MSL and RB and between MSI and RB. Therefore, the following hypotheses were made:

***H3: HS has a positive moderating effect on the relationship between MSL and*** ***RB, as well as the relationship between MSI and RB*.**

Studies show that the migrants prevalently have a low health literacy. Due to a lack of health-related knowledge, migrants are more likely to resort to self-care when sick than seek medical care (37). At the same time, when faced with a major illness, people with poor health literacy may delay seeking medical care because they are unaware of preventive measures or symptoms of the illness, which may eventually lead to the deterioration of the condition (38). HE refers to well-planned, organized, systematic social education activities aiming to help people voluntarily adopt behaviors and lifestyles beneficial for their health (28). HE is considered an important path toward improving health literacy among the migrants (39). The higher the level of HE received by the migrants, the higher their health awareness and their need for medical services. That is, HE strengthens their attention to medical services. Therefore, when the MSL is low but the MSI continues to grow, the migrants with higher HE levels is more likely to choose to return to the city with household registration, pointing to the positive moderating effect of HE.

***H4: HE has a positive moderating effect on the relationship between MSL and*** ***RB, as well as the relationship between MSI and RB*.**

## Methodology

### Data source

The data in this study are mainly derived from the CMDS in 2017 and the China City Statistical Yearbook in 2018. CMDS, released by the National Health Care Commission, is based on the randomization principle of screening and locking sample sites in the more concentrated areas of the migrants in 31 provinces (Districts, Cities) and the Xinjiang Production and Construction Corps in mainland China, while using a stratified, multi-stage, size-proportional PPS method for sampling. The target respondents of the database are the migrants who have lived in the inflow area for more than 1 month and are 15 years old and above, with a sample size of about 170,000^2^. Detailed information on the purpose, design, sample and questionnaire of the CMDS can be found in the CMDS-related design scheme published by the National Health Commission. City-level data were obtained from the China City Statistical Yearbook published by the National Bureau of Statistics. In this paper, we determined whether the return behavior of the migrants occurred: using the prefecture-level city as the unit, the prefecture-level city to which the sample belonged was matched with the city-level data. After excluding missing values, the total sample size is 111,540.

### Measurement

#### Dependent variables

Unlike previous studies on migration intentions, this paper was based on the results of the CMDS survey to screen samples in a two-step process to determine whether the respondents' actual RB occurred. In the first step, if the respondents had left the place of household registration for 6 months or more, and their permanent residence was the same as the place of household registration at the time of the survey, the preliminary decision was returned (23). In the second step, according to question 304.1 of the CMDS questionnaire, the samples whose flow range was “intra-city flow” were excluded. Ensure that the respondents left the city where their household registration was in the flow, which was in line with the definition of return behavior in this paper.

#### Independent variables

According to the purpose of the study, MSL and MSI were used as independent variables. Drawing on Li's definition of medical service and based on data from the China City Statistical Yearbook in 2018 (40, 41), this paper selected a total of five indicators, namely the number of hospitals, the number of hospital beds, the number of licensed physicians, the number of urban workers' basic old-age insurance participants, and the number of urban workers' basic medical insurance participants. Factor analysis in principal component analysis was used to weight the composite score as MSL in the city where the migrants is located. This paper considered MSI as the improvement degree of MSL, which can be regarded as the MSL of a city in a certain year minus the MSL of 6 years ago. Specifically, through principal component analysis, each city's comprehensive scores of MSL in 2017 and 2012 were separately calculated, and the former minus the latter was used as MSI of the city where the survey respondent was located.

#### Moderator variable

The moderator variables in this study were HS and HE. According to the Health and Public Services survey module of CMDS data, respondents were required to fill in the question, “whether you had any illness or physical discomfort in the last year?” If the answer was yes, the code was counted as 1, defining that in poor HS; if the answer were no, the code was counted as 0, defining that in good HS. In the CMDS, respondents were asked to answer the nine HE questions, “In the past year, did you receive health education on mental health/smoking/chronic diseases/AIDS/occupational diseases/maternal and child health/tuberculosis/reproductive health and contraception/self-help in public emergencies?” Participation in one of these HE was counted as 1, not counted as 0, and the score ranges 0–9.

#### Control variables

To obtain the effects of medical services on RB of migrants, this paper controlled other factors that may affect it. Based on the control variable designs of existing scholars (23, 28), this paper selected control variables from the city and individual levels. From the city level, five variables were selected: gross regional product per capita (GRP), education status (ES), region attribution (RA), logistics infrastructure (LI), and information infrastructure (II). From the individual level, a total of five variables were selected: age characteristics (AC), RHR, NRCMS, educational background (EB), and personal income (PI). The measurement of control variables is shown in Table 1.

**Table 1:** Control variable measurement.

**Variable name**	**Variable symbol**	**Description**
Gross regional product per capita	GRP	The amount of GRP per capita in the city in which the migrants currently resided in 2017.
Education status	ES	The number of primary and secondary school students in the city where migrant workers were located in 2017.
Region attribution	RA	According to the regional division of the city to which the migrants belong, the RA variable was divided into three groups: eastern region, central region, and western region.
Logistics infrastructure	LI	Revenue from the city's postal business at year-end.
Information infrastructure	II	Revenue generated by the city's telecommunications business at year-end.
Age characteristics	AC	Variables derived from CMCD data were used to distinguish migrants by their age stage.
Rural household registration	RHR	Data from the CMDS was used to determine whether a respondent was an agricultural registered permanent resident. If the answer was yes, the code was counted as 1, not counted as 0.
New rural cooperative Medical system	NRCMS	Survey respondents about their participation in this rural health insurance program using CMDS data^3^. Participation in this insurance was counted as 1, not counted as 0.
Educational background	EB	Accordingly to the statistics of the educational background of CMDS data, primary schools and below were counted as 1, junior high schools, vocational colleges, and senior high schools were counted as 2, undergraduates and junior colleges were counted as 3, and graduates and above were counted as 4.
Personal income	PI	Based on data from the CMDS survey, the average monthly income of the respondent in the past year was used.

### Empirical model

Considering that the explained variable RB is a binary variable, Probit model is suitable for modeling. Equations (A)~(D) verify four research hypotheses, respectively:


(A) $$\begin{array}{l} Prob\left(RB_{ij}=1|MSL_{j},MSI_{j},HS_{ij},HE_{ij},control_{1,ij},...,control_{M,ij}\right)\\ =\Phi \left(\beta_{0}+\beta_{1}MSL_{j}+\beta_{2}MSI_{j}+\underset{m=1,...,M}{\sum }\beta_{m+3}control_{m,ij}+\varepsilon_{ij}\right) \end{array}$$

$$\begin{array}{l} Prob\left(RB_{ij}=1|MSL_{j},MSI_{j},HS_{ij},HE_{ij},control_{1,ij},...,control_{M,ij}\right)\\ =\Phi (\beta_{0}{+\beta }_{1}MSL_{j}+\beta_{2}MSI_{j}{+\beta }_{3}MSL_{j}\times MSI_{j} \end{array}$$

(B) $$\begin{array}{l} +\underset{m=1,...,M}{\sum }\beta_{m+3}control_{m,ij}+\varepsilon_{ij}) \end{array}$$

$$\begin{array}{l} Prob(RB_{ij}=1|MSL_{j},MSI_{j},HS_{ij},HE_{ij},\\ control_{1,ij},...,control_{M,ij}) \end{array}$$

(C) $$\begin{array}{l} =\Phi \left(\begin{array}{c} \beta_{0}+\beta_{1}MSL_{j}+\beta_{2}MSI_{j}+\beta_{3}{HS_{ij}\times MSL}_{j}\\ +\beta_{4}HS_{ij}\times MSI_{j}\\ +\underset{m=1,...,M}{\sum }\beta_{m+4}control_{m,ij}+\varepsilon_{ij} \end{array}\right) \end{array}$$

$$\begin{array}{l} Prob(RB_{ij}=1|MSL_{j},MSI_{j},HS_{ij},HE_{ij},\\ control_{1,ij},...,control_{M,ij}) \end{array}$$

(D) $$\begin{array}{l} =\Phi \left(\begin{array}{c} \beta_{0}+\beta_{1}MSL_{j}+\beta_{2}MSI_{j}+\beta_{3}{HE_{ij}\times MSL}_{j}\\ +\beta_{4}HE_{ij}\times MSI_{j}\\ +\underset{m=1,...,M}{\sum }\beta_{m+4}control_{m,ij}+\varepsilon_{ij} \end{array}\right) \end{array}$$

Taking Equation (A) as an example, it represents the probability that *i* (migrants) chooses to return from *j* (inflow area) under given MSL, MSI and other conditions. Moreover, *control*_*ij*_ represents the control variable, and ε_*ij*_ is the residual term. Equations (B)~(D) are based on Equation (A), and the other variables have the same meaning.

## Results

### Factor analysis

The explanatory variable MSL was measured by calculating the composite score using factor analysis in principal component analysis. In this paper, the composite scores of MSL in 2017 and 2012 were calculated in turn. By performing the KMO test and Bartlett's sphericity test on the indicators, it was found that the KMO values were 0.793 and 0.803, respectively, and the significance of Bartlett's sphericity test was at the 1% level, indicating that the above data were suitable for the factor analysis method. Most public factor extraction degrees were above 0.8, indicating a good information extraction effect. According to the criterion that the characteristic root is >1 to extract the common factors, the variance contribution rate is 88.70% and 83.99%, which can better represent most of the information of the original indexes. The score coefficient matrix derived from the factor rotation using the maximum variance method was combined with the score coefficient matrix using the variance contribution rate as the weight to calculate the composite score. The variable MSL was measured using the 2017 composite score, and the variable MSI was measured using the difference between the 2017 composite score and 2012 for the following statistics and tests.

### Descriptive statistics

In order to see more clearly the differences in RB among the migrants, the sample was divided into two groups for descriptive statistics based on the occurrence or non-occurrence of RB. The variables for all descriptive statistics are shown in Table 2.

**Table 2:** Descriptive statistics.

**Variables**	**RB**	**Mean**	**SD**
MSL	Occurrence	−0.54	0.37
	Non-occurrence	0.03	1.01
MSI	Occurrence	−0.07	0.19
	Non-occurrence	0.01	0.27
HS	Occurrence	0.46	0.50
	Non-occurrence	0.45	0.50
HE	Occurrence	3.83	3.48
	Non-occurrence	3.32	3.39
GRP	Occurrence	5.65	2.87
	Non-occurrence	8.61	3.65
ES	Occurrence	67.18	37.99
	Non-occurrence	84.87	74.17
RA	Occurrence	2.04	0.61
	Non-occurrence	1.78	0.83
LI	Occurrence	15.30	23.62
	Non-occurrence	48.51	66.51
II	Occurrence	44.11	38.21
	Non-occurrence	139.43	177.78
AC	Occurrence	35.95	8.86
	Non-occurrence	37.55	10.98
RHR	Occurrence	0.78	0.42
	Non-occurrence	0.78	0.42
NRCMS	Occurrence	0.71	0.45
	Non-occurrence	0.79	0.41
EB	Occurrence	2.56	0.94
	Non-occurrence	2.38	0.97
PI	Occurrence	0.67	0.48
	Non-occurrence	0.71	0.55

As can be seen from Table 2, from the perspective of cities, the difference in MSL variables in the two groups is obvious. The average value of the non-occurrence group of RB is much higher than that of the occurrence group, indicating that the current migrants that does not return is generally located in areas with abundant medical resources, which is also in line with the fact that the current migrants generally go to work in coastal and developed areas of China. However, the difference in the variable of MSI between the two groups is small, indicating that with the continuous improvement of China's medical service system, medical services have gradually covered less developed and remote areas, leading to a further increase in the equality of medical services among cities in China. In terms of LI, II, GRP per capita, and ES, there is a huge gap in economic development, educational resources, logistics construction and information construction between the two groups. Moreover, from the individual level, the variable of HE was significantly different between the occurrence group and the non-occurrence group, which indicates that the migrants with RB generally received a higher degree of HE. Except for the HE variable, there was no significant difference in other variables.

### Regression result

Stepwise Probit regression was performed after data standardization for continuous variables, the LR CHI2 values of the Models (A)~(D) were all significant in Table 3, indicating that the overall regression effect of each model was good.

**Table 3:** Results of Probit regression.

**Variables**	**RB**			
	**Model (A)**	**Model (B)**	**Model (C)**	**Model (D)**
MSL	−0.27^***^ (-6.92)	−0.43^***^ (-11.03)	−0.29^***^ (-7.16)	−0.29^***^ (-7.14)
MSI	0.18^***^ (4.60)	0.00 (0.05)	0.12^**^ (2.39)	0.03 (0.45)
MSL × MSI		0.86^***^ (27.91)		
HS	−0.17^***^ (-12.74)	−0.16^***^ (-11.58)	−0.13^***^ (-6.95)	−0.17^***^ (-12.63)
HE	0.01^***^ (5.40)	0.01^***^ (3.84)	0.01^***^ (5.48)	0.02^***^ (6.06)
MSL × HS			0.06^**^ (2.34)	
MSI × HS			0.16^**^ (2.32)	
MSL × HE				0.01^**^ (2.05)
MSI × HE				0.04^***^ (3.88)
GRP	−0.18^***^ (-16.15)	−0.15^***^ (-12.67)	−0.18^***^ (-16.19)	−0.18^***^ (-16.24)
ES	0.52^***^ (25.49)	0.71^***^ (33.47)	0.52^***^ (25.08)	0.52^***^ (25.31)
RA	0.05^***^ (4.86)	0.06^***^ (5.56)	0.05^***^ (5.00)	0.05^***^ (4.71)
LI	0.30^***^ (12.68)	−0.14^***^ (-5.27)	0.31^***^ (12.98)	0.31^***^ (12.94)
II	−2.02^***^ (-28.38)	−1.65^***^ (-23.11)	−2.02^***^ (-28.42)	−2.02^***^ (-28.46)
AC	−0.05^***^ (-7.08)	−0.05^***^ (-6.39)	−0.05^***^ (-7.06)	−0.05^***^ (-7.04)
RHR	−0.07^***^ (-3.77)	−0.08^***^ (-3.88)	−0.07^***^ (-3.75)	−0.07^***^ (-3.71)
NRCMS	0.16^***^ (9.03)	0.12^***^ (6.99)	0.16^***^ (9.08)	0.15^***^ (9.00)
EB	0.14^***^ (17.37)	0.14^***^ (17.40)	0.14^***^ (17.40)	0.14^***^ (17.44)
PI	0.01^**^ (1.97)	0.01^*^ (1.87)	0.01^**^ (1.99)	0.01^*^ (1.91)
Pseudo R2	0.16	0.17	0.16	0.16
LR CHI2	8081.69^***^	8956.26^***^	8090.58^***^	8098.36^***^

The numbers in parentheses are Z-test values.

***, **, and * represent significance at the 1, 5, and 10% levels, respectively. The following are the same.

As can be seen from Model (A) in Table 3, after controlling for other variables that may affect the RB of migrants, MSL has a significant negative impact on RB of migrants (β = −0.27, *p* < 0.01), and the MSI has a significant positive impact on RB of migrants (β = 0.18, *p* < 0.01). It indicates that the RB of migrants tends to the original household registration cities with relatively low MSL but rapid MSI. Hypothesis 1 has been verified.

On the basis of Model (A), Model (B) added the intersection term of MSL and MSI. It can be seen from Model (B) that after controlling for other variables that may affect RB, MSL and MSI are not significantly different from Model (A), and MSL × MSI has a significant positive impact on RB of migrants (β = 0.86, *p* < 0.01). It indicates that MSL and MSI play a positive interaction effect, which jointly increases the probability of RB. Hypothesis 2 is verified.

On the basis of Model (A), Model (C) added the intersection terms of MSL and HS, as well as MSI and HS. As shown from Model (C), after controlling for other variables that may affect RB, MSL and MSI, there is no significant difference with Model (A). At the same time, MSL × HS and MSI × HS have a significant positive influence on RB (β = 0.06, *p* < 0.05; β = 0.16, *p* < 0.05), indicating that HS play a positive moderating role, and hypothesis 3 is verified.

On the basis of Model (A), the intersection terms of MSL and HE, MSI and HE are added in Model (D). As you can see in Model (D), after controlling for other variables that may affect RB, MSL and MSI, there is no significant difference with Model (A). At the same time, MSL × HE and MSI × HE have a significant positive influence on RB (β = 0.01, *p* < 0.05; β = 0.04, *p* < 0.01), indicating that HE play a positive moderating role, and hypothesis 4 is verified.

### Heterogeneous impact analysis

Considering that most migrants in the Chinese scenario have household registration in rural areas (7), their physical health and medical behavior are also widely concerned (36, 38). This study considers the different performances of rural migrants and non-rural migrants in the decision-making process of return. Therefore, it is necessary to distinguish the rural migrants for more in-depth analysis and exploration. In order to better screen out the rural migrants from the original migrant sample, the control variables RHR and NRCMS, which reflect the rural attributes of migrants, were selected for grouping. Based on Model (A) in Table 2, Models 1a and 1b are constructed according to whether migrants are agricultural registered permanent residents, and Models 1c and 1d are constructed based on whether the migrants have NRCMS insurance. The results of Probit regression analysis for Models 1a~1d are shown in Table 4.

**Table 4:** Results table of Probit regression for different groups.

**Variables**	**RB**			
	**Model 1a**	**Model 1b**	**Model 1c**	**Model 1d**
	**RHR**	**Non-RHR**	**NRCMS**	**Non-NRCMS**
MSL	−0.21^***^ (-4.74)	−0.53^***^ (-7.04)	−0.15^***^ (-3.07)	−0.35^***^ (-5.72)
MSI	0.24^***^ (5.51)	−0.06 (-0.65)	0.07 (1.40)	0.29^***^ (3.72)
HS	−0.17^***^ (-11.22)	−0.16^***^ (-5.44)	−0.20^***^ (-12.21)	−0.12^***^ (-4.80)
HE	0.01^***^ (4.60)	0.01^**^ (2.42)	0.01^***^ (3.20)	0.02^***^ (4.98)
GRP	−0.19^***^ (-15.00)	−0.17^***^ (-6.83)	−0.21^***^ (-14.76)	−0.14^***^ (-7.46)
ES	0.53^***^ (22.75)	0.52^***^ (11.43)	0.58^***^ (23.78)	0.41^***^ (10.55)
RA	0.08^***^ (6.84)	−0.04^**^ (-1.99)	0.08^***^ (6.51)	−0.01 (-0.39)
LI	0.30^***^ (11.60)	0.32^***^ (5.78)	0.29^***^ (8.63)	0.28^***^ (7.92)
II	−2.10^***^ (-25.51)	−1.62^***^ (-11.46)	−2.31^***^ (-24.81)	−1.51^***^ (-14.02)
AC	−0.04^***^ (-4.40)	−0.09^***^ (-5.60)	−0.05^***^ (-5.74)	−0.05^***^ (-3.60)
RHR			−0.20^***^ (-6.72)	−0.01 (-0.45)
NRCMS	0.11^***^ (5.71)	0.22^***^ (6.49)		
EB	0.16^***^ (17.58)	0.08^***^ (4.94)	0.16^***^ (15.19)	0.12^***^ (9.16)
PI	0.01 (1.11)	0.03^**^ (2.19)	0.01 (0.95)	0.02^*^ (1.94)
Pseudo R^2^	0.16	0.15	0.17	0.14
LR CHI2	6510.30^***^	1681.55^***^	6099.88^***^	2144.43^***^
Number	88313	23227	73264	38276

The numbers in parentheses are Z-test values.

***, **, and * represent significance at the 1, 5, and 10% levels, respectively.

In Models 1a and 1b of Table 4, there is a heterogeneous effect of MSL and MSI on RB under different household registration nature. The coefficients of MSL and MSI in the RHR subgroup are larger and both significant, indicating that for the rural migrants, it is more probable to return to the city of their household registration where MSL is lower but MSI is faster. The regression results from models 1c and 1d show that the coefficients of MSL and MSI are larger for the Non-NRCMS subgroup and both are significant. The findings suggest that for the migrants not covered by basic medical insurance, they are more likely to choose to return for more economical medical services in the city of their household registration. The reason for the difference is that the introduction of the NRCMS has promoted the willingness of the rural population to see a doctor, but the burden of medical expenses of the rural household has further increased. Yao et al. (42) conducted a study on urban-rural medical disparities in China and found that medical insurance holders in agricultural households have higher out-of-pocket costs and hospitalization costs than non-agricultural households and are also more likely to be induced by medical treatment to incur more costs. Thus, considering the high health care burden in the inflow area and the cumbersome reimbursement process of the NRCMS, both for the migrants with household registration in a rural area and for the uninsured migrants, they are influenced by the city-level medical services and thus are more likely to make the decision to return to their hometown.

In order to further refine the effects of MSL and MSI on RB, this paper is based on the dual grouping of migration distance and age, inspired by Gu et al. (19) view that “health services affect the migration distance of older people.” Migration distance and age are also from CMCD data. The heterogeneity analysis of the sample can explain to some extent the difference in the decision to return between middle-aged and older people and younger people. Referring to the WHO age classification criteria, this paper takes 45 years as the cut-off, and samples below this age are young people, while others are middle-aged and older people. Then the migration distance is distinguished according to inter-provincial migration and intra-provincial migration, so the samples are divided into middle-aged and older people who return across provinces (IREG), middle-aged and older people who return within provinces (PREG), young people who return across provinces (IRYG), young people who return within provinces (PRYG), and those who do not return. Model 2a~2d were the experimental groups, and Model 2e participated in the regression as the baseline group. The results of multinomial Probit regression analysis for Models 2a~2e are shown in Table 5.

**Table 5:** Results table of multinomial Probit regression for different groups.

**Variables**	**RB**				
	**Model 2a**	**Model 2b**	**Model 2c**	**Model 2d**	**Model 2e**
	**IREG**	**PREG**	**IRYG**	**PRYG**	**Non-return population**
MSL	0.04 (0.33)	−0.65^***^ (-10.21)	−0.36^**^ (-2.23)	−0.04 (-0.42)	
MSI	0.64^***^ (6.02)	0.36^***^ (5.46)	−0.09 (-0.57)	−0.29^***^ (-3.39)	
HS	−0.09^**^ (-2.45)	−0.25^***^ (-11.31)	−0.18^***^ (-3.68)	−0.24^***^ (-8.41)	
HE	0.02^***^ (3.70)	0.02^***^ (4.77)	0.01^*^ (1.72)	0.01 (1.45)	
GRP	−0.32^***^ (-9.37)	−0.25^***^ (-13.24)	−0.11^***^ (-2.76)	−0.19^***^ (-7.91)	
ES	0.57^***^ (10.69)	0.82^***^ (24.53)	0.50^***^ (6.50)	0.53^***^ (12.15)	
RA	0.11^***^ (3.78)	0.11^***^ (6.51)	0.01 (0.32)	−0.06^***^ (-2.96)	
LI	0.38^***^ (5.28)	0.35^***^ (8.37)	0.37^***^ (3.84)	0.52^***^ (9.80)	
II	−2.61^***^ (-12.36)	−2.28^***^ (-19.82)	−2.28^***^ (-8.13)	−3.34^***^ (-19.52)	
RHR	−0.16^***^ (-2.89)	0.02 (0.63)	−0.37^***^ (-5.44)	−0.11^***^ (-2.89)	
NRCMS	0.09^*^ (1.81)	0.32^***^ (11.00)	0.05 (0.87)	0.14^***^ (3.90)	
EB	−0.19^***^ (-8.43)	0.22^***^ (17.75)	−0.12^***^ (-3.89)	0.45^***^ (28.03)	
PI	−0.01 (-0.30)	0.04^***^ (3.37)	0.03 (1.13)	−0.03^*^ (-1.73)	
WALD CHI2			6085.83^***^		
Number	784	3,888	337	1,791	104,740

The numbers in parentheses are Z-test values.

***, **, and * represent significance at the 1, 5, and 10% levels, respectively.

Models 2a~2d are regression results based on return distance and age grouping, as shown in Table 5. The results show that the coefficients of MSL indicator in the four models are 0.04,−0.65,−0.36, and−0.04, indicating that the tendency degree of return because of MSL is ranked as IREG> PRYG> IRYG> PREG. The coefficients of MSI indicator in the four models are 0.64, 0.36,−0.09, and−0.29, indicating that the tendency degree of return because of MSI is ranked as IREG> PREG> IRYG> PREG. The results suggest that PREG chooses to return when MSL is low and MSI is high in the city with household registration, while IREG tends to choose to return when MSI is high and is likely to return when MSL is high. Compared to middle-aged and older people, PRYG are least likely to return because of MSL and MSI of the city of household registration. PRYG still choose to return when MSL is low, but are not very attached to MSI.

By comparing the MSL results of each group in Table 5, it can be seen that the level of medical services in IREG is not significant compared to PREG, indicating that the need for medical services is more urgent for the elderly returning across provinces. Due to more restrictions on inter-province medical services, middle-aged and older adults tend to return across provinces even though higher MSLs in the city of household registration can result in higher medical costs. In comparison, PREGs are closer in migration distance and can reach the city of household registration in a short time. Furthermore, the interoperability of the provincial health care system reduces certain medical costs, so they do not tend to return to the city of household registration when MSL is higher. However, for young people, PRYG is not significant compared to IRYG. These findings are understandable because young people have a lower demand for medical services compared to middle-aged and older people, and more often go to work outside the province for reasons such as salary and development opportunities. Young people who return to the province are less disturbed by the level of medical services, so the empirical result is insignificant. The MSI analysis of each subgroup shows that both IREG and PREG are positively significant, indicating that for middle-aged and older people, the improvement of MSLs in the household registration city has a strong attraction to return and can meet their medical needs. The MSI coefficients of IRYG and PRYG are negative, indicating that the return attraction brought by the improvement of medical services is insufficient for young people. Moreover, young people may stay in the outflow city and not choose to return due to other considerations.

## Discussion

With the emergence of the trend of return migration in China and the deepening of the reform of the household registration system since the 1990s, many disciplines such as geography, economics, sociology, and demography have paid attention to the issue of return migration. While early studies focused on the demographic and socio-economic characteristics of migrants, in recent years, the initiative and selectivity of migrants have received more attention. This paper focuses on the phenomenon of migrants choosing to return due to medical services, and has important practical implications for local governments to improve the level of medical services, increase the growth rate of medical services, pay attention to the medical needs of the elderly, and reduce the gap in medical services between urban and rural areas. On the whole, the paper has the following contributions.

Firstly, in this study, we explored medical services effects on RB. The current research on return migration is mostly about return intentions. Although it predicts the decision-making tendency of migrants to a certain extent, whether RB occurs is unknown (20). It was notable that the survey of migrants also found that many people with return intentions did not choose to return due to family, work, medical treatment, education and other reasons. This paper uses the large sample data of CMDS to identify whether the exact RB of migrants occurs, defines the RB of migrants from the perspective of spatial flow, and also provides reference for the subsequent research on RB. Furthermore, this paper divided the medical service into two aspects: MSL and MSI, which expanded the connotation of the concept of medical service. MSL represents the capacity and strength of the current medical service, while MSI represents the development potential and growth trend. It was found that low MSL and large MSI in the city of household registration would increase the return possibility of migrants. In addition, MSL and MSI have a positive interaction effect on RB. Due to the limitations of the household registration system and economic situation, the migrants do not blindly pursue the absolute high MSL but chooses their hometown with greater development potential for medical services. Accordingly, the phenomenon is worth further thinking about. Undeniably, this study not only deepens the RB of migrants under the influence of medical services, expands the scope of return research, but also provides practical value for local governments to grasp the psychological needs of migrants for medical services, pay attention to the steady and rapid medical services, and attract more migrants to return to the city of their household registration.

The second, we explored the moderating effects of HS and HE on medical services and RB. We found that HS and HE has positive moderating effects on the relationship between MSL and RB and the relationship between MSI and RB, respectively. Whether it is MSL or the MSI, the premise of the impact on the RB of migrants is that the migrant people need certain medical services, but these medical service needs vary from person to person. In order to further explore the mechanism of medical services on RB under different scenarios, two health factors of migrants were selected as moderating variables from the perspective of medical service demand. Previous studies have shown that HS and HE are both important factors affecting medical services of migrants (32, 39), scenario-based studies using the two as moderating variables have strong theoretical and practical significance.

Then, we selected two variables for heterogeneity analysis separately. Grouping regressions were performed on the two variables, RHR and NRCMS. It was notable that the RHR group or the Non-NRCMS group are more likely to return as a result of the level and improvement of medical services. Some scholars have paid attention to the huge difference in medical services between urban and rural populations. Studies have pointed out that the rural population generally has problems such as weak health awareness, limited health investment, high medical expenses, and low utilization of medical services (42). In this case, the migrants with RHR will have more restrictions on medical services. At the same time, the NRCMS, as a government-organized mutual assistance system for rural residents, is unable to better enjoy medical services in the inflow area due to the restriction of reimbursement from other places so as to reduce the medical burden. The above analysis shows the disadvantages of the current rural migrants in medical services, and inspires the government to pay attention to the coordinated development of regional medical services.

Finally, the age and migration distance factors of the migrants were considered. The multinomial group regression was carried out. It was found that middle-aged and older people with long migration distances chose to return when both MSL and MSI were higher, and those with short migration distances chose to return when MSL was lower and MSI was higher. In contrast, young people have a lower overall willingness to return for medical services regardless of migration distance. From the above analysis, one may conclude that the migrants of different ages have different tendencies to return due to the factors of medical services under the limitation of different migration distances. Therefore, under the multinomial grouping of migration distance and age, the paper makes a deep interpretation of the conclusion of the relationship between medical services and RB in different scenarios.

This study still has some limitations and shortcomings. On the one hand, medical services involve many aspects. This study only selects MSL and MSI from the city level and does not explore other aspects of medical services. On the other hand, panel data was not used for analysis. Given the randomness of a questionnaire survey, it is less likely that the same person will be surveyed for several consecutive years. Therefore, the current mainstream studies on migrants all use cross-sectional data. However, cross-sectional data have some flaws in the long-term migration behavior of the migrants. In the future, data from other sources will be considered for detailed research.

## Conclusion

Using data from CMDS as a sample, medical services were separated into two aspects: MSL and MSI, and the relationship between medical services and RB was explored using Probit regression analysis to investigate the interaction effects of MSL and MSI, as well as the moderating effects of HS and HE. Moreover, the migrants were further regressed in the heterogeneity analysis by grouping them separately according to whether they possessed RHR or whether they participated in NRCMS. Equally, multinomial Probit regression was conducted for migrants according to whether they were middle-aged and older people and whether they were interprovincial migrants.

(1) When MSL is low but the MSI is large, the return probability of migrants will increase. There is also a positive interaction effect between MSL and MSI. The larger the interaction effect, the higher the return probability of migrants.
(2) HS and HE have a positive moderating effect on the relationship between MSL and RB, as well as the relationship between MSI and RB.
(3) Through heterogeneity analysis, this paper shows that migrants with RHR and those who did not participate in the NRCMS were more likely to choose to return due to medical services. The analysis also shows that middle-aged and older people who return across provinces have the highest tendency to return due to medical services, and young people have the lowest tendency both across and within provinces.

Local governments should pay attention to the groups of migrants and the supply of medical service resources and grasp the real attractiveness of medical services implied by the status quo of the household registration system and market demand. The study has the following insights:

(1) First, the government needs to focus on improving MSL and increasing the economic support for basic medical services. To achieve full coverage of medical insurance and comprehensive reform of urban public hospitals, promote the national networking of basic medical insurance and settlement of medical treatment in different places, and better realize the allocation of medical resources for the migrants. Especially for the regions with backward MSL, it is more necessary to try to tap medical service resources to enhance medical service upgrading potential to really narrow the gap with well-developed regions.
(2) Secondly, regarding population migration management, the government should pay attention to the HS and HE of migrants and strengthen the health records of the migrants. The government should actively carry out health education-related publicity work, so that the migrants can, as far as possible, prevent problems before they occur and seek medical treatment in time to avoid minor illnesses becoming serious ones. It is necessary to update the health records of the migrants on time and accelerate the sharing of information. The government should ensure the authenticity, integrity, and scientific nature of the information in the health records of the migrants and gradually realize the interconnection of the health records of the migrants across regions and departments.
(3) Finally, the government should pay attention to the medical service needs of the rural household migrants and promote the reform of the rural medical system. It should also strengthen the construction of the rural medical service system and eliminate the gap areas in rural medical and health institutions. At the same time, the government should care for the middle-aged and older people and help them obtain the same or similar medical coverage as the registered elderly population in the inflow area. Communities can establish service centers for the elderly based on the service and management of the migrants in their jurisdictions and provide relevant services directly to the middle-aged and older people.

## Data availability statement

The original contributions presented in the study are included in the article/supplementary material, further inquiries can be directed to the corresponding author.

## Author contributions

MM and JY developed the main ideas of the study, gathered the data, performed the model construction and estimation, and wrote the manuscript. ZW participated in revising the manuscript and proofreading the article. All authors have read and agreed to the published version of the manuscript.

## Funding

This article was supported by the project study on influencing factors of labor productivity (#KY-2020-wt010) from Chinese Academy of Labour and Social Security.

## Conflict of interest

The authors declare that the research was conducted in the absence of any commercial or financial relationships that could be construed as a potential conflict of interest.

## Publisher's note

All claims expressed in this article are solely those of the authors and do not necessarily represent those of their affiliated organizations, or those of the publisher, the editors and the reviewers. Any product that may be evaluated in this article, or claim that may be made by its manufacturer, is not guaranteed or endorsed by the publisher.

## Footnotes

^1^Considering that the sample is 2017 data, the paper analyzes the problems of medical treatment in other places mainly for the scenario in 2017. However, as of early 2022, there are still more problems, such as cumbersome direct settlement filing procedures, different settlements and reimbursement policies between regions, and unconnected information systems for heterogeneous medical treatment.

^2^Both “inflow” and “outflow” data are obtained from the CMCD, with “inflow” representing the current city of the survey respondent and “outflow” representing the city of the household registration of the survey respondent before the migration occurred. If the CMCD data for a specific survey respondent shows that the “inflow” city and the “outflow” city are the same, it means that a return behavior has occurred after the migration. In this paper, the return behavior is defined as the phenomenon that the migrant population leaves the city of their household registration and returning to their city after a period of time, i.e., for the returning population, both “inflow” and “outflow” represent the city where the household registration is located, and they are the same.

^3^In January 2016, the State Council issued “The Opinions on Integrating Urban and Rural Residents Basic Medical Insurance System,” deciding to integrate the two systems of urban residents' medical insurance and NRCMS into a unified basic medical insurance system for urban and rural residents, but as of 2017 the opinion is still in the promotion stage, and CMDS data show that in 2017 the migrant population is still mainly participating in the NRCMS.
